# Sense of external agency is sustained by multisensory functional integration in the somatosensory cortex

**DOI:** 10.1002/hbm.25107

**Published:** 2020-07-15

**Authors:** Federica Piras, Daniela Vecchio, Valentina Ciullo, Tommaso Gili, Nerisa Banaj, Fabrizio Piras, Gianfranco Spalletta

**Affiliations:** ^1^ Department of Clinical and Behavioral Neurology, Neuropsychiatry Laboratory IRCCS Santa Lucia Foundation Rome Italy; ^2^ Networks Unit, IMT School for Advanced Studies Lucca Italy; ^3^ Menninger Department of Psychiatry and Behavioral Sciences Baylor College of Medicine Houston Texas USA

**Keywords:** healthy subjects, primary somatosensory area, psychiatric disorders, rs‐fMRI, self‐concepts, sense of agency, visual cortex

## Abstract

“Sense of agency” (SoA), the feeling of control for events caused by one's own actions, is deceived by visuomotor incongruence. Sensorimotor networks are implicated in SoA, however little evidence exists on brain functionality during agency processing. Concurrently, it has been suggested that the brain's intrinsic resting‐state (rs) activity has a preliminary influence on processing of agency cues. Here, we investigated the relation between performance in an agency attribution task and functional interactions among brain regions as derived by network analysis of rs functional magnetic resonance imaging. The action‐effect delay was adaptively increased (range 90–1,620 ms) and behavioral measures correlated to indices of cognitive processes and appraised self‐concepts. They were then regressed on local metrics of rs brain functional connectivity as to isolate the core areas enabling self‐agency. Across subjects, the time window for self‐agency was 90–625 ms, while the action‐effect integration was impacted by self‐evaluated personality traits. Neurally, the brain intrinsic organization sustaining consistency in self‐agency attribution was characterized by high connectiveness in the secondary visual cortex, and regional segregation in the primary somatosensory area. Decreased connectiveness in the secondary visual area, regional segregation in the superior parietal lobule, and information control within a primary visual cortex‐frontal eye fields network sustained self‐agency over long‐delayed effects. We thus demonstrate that self‐agency is grounded on the intrinsic mode of brain function designed to organize information for visuomotor integration. Our observation is relevant for current models of psychopathology in clinical conditions in which both rs activity and sense of agency are altered.

## Graphical abstract

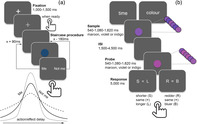

We investigated the relation between agency and functional interactions among brain regions using network analysis. Self‐agency is grounded on the intrinsic mode of brain function designed to organize information for visuomotor integration. This is relevant for current models of psychopathology in clinical conditions in which both brain functional activity and sense of agency are altered.

## 1 INTRODUCTION

The prevalent definition of *sense of agency* (SoA), that is, the feeling that we are intentionally making things happen by our own action (Synofzik, Vosgerau, & Newen, 2008b), encapsulates the two layers of such experience: that over the performed actions (i.e., *body agency*) and that over external events (i.e., *external agency*) (Farrer & Frith, 2002). Body agency emerges from a low‐level implicit sensorimotor integration between motor commands and sensory feedbacks regarding the body. Though, external agency is based on spatial and temporal tight contiguity between actions and effects enabling the learning of causal relations between one's behavior and environmental changes. Further high‐level conceptual processing form an *attribution judgment of agency* (JoA) integrating context cues, background beliefs, and *post‐hoc* inferences, as to ascribe an action to the self's or somebody else's agency (Synofzik, Vosgerau, & Newen, 2008a).

The comparator view model (Blakemore, Wolpert, & Frith, 2002; Frith, Blakemore, & Wolpert, 2000; Wolpert & Flanagan, 2001), which dominated research on SoA for decades, essentially proposes that JoA is generated by comparing a predicted outcome of intended motor plans to the actual proprioceptive/visual feedback information (i.e., sensorimotor integration). Accordingly, two types of internal models are implemented in the central motor system to control and optimize motor behavior: the inverse model to determine the motor commands necessary to achieve a desired state, and forward models to allow the system to predict the expected sensory feedback of a motor command. The sense of agency particularly hinges on the forward model, which uses an efference copy, that is, a copy of a motor command predicting respective sensory consequences (Blakemore et al., 2002; Wolpert & Flanagan, 2001). When the prediction is congruent with the actual outcome, then agency is attributed to the self (i.e., *self‐agency attribution*) (Gallagher, 2007); if not, then agency is attributed elsewhere (Frith et al., 2000). To form an attribution JoA, a precise intentional content is needed followed by an effect that resembles this content. Information about the operation of intermediate mechanisms that select the proper course of action to achieve the desired effect is then used to causally relate intentions to subsequent actions (Chambon & Haggard, 2013).

The multifactorial weighting model (Synofzik et al., 2008a), going beyond sensorimotor integration defined in the comparator view model, decomposes the SoA into agency determination through the motor control system (Chambon & Haggard, 2013) and by more conceptual processes. This last model defines that many different agency cues are constantly weighted according to their reliability in a given situation (Moore & Fletcher, 2012; Vosgerau & Synofzik, 2012). The importance of the different authorship signals varies depending on task, context and person (Synofzik et al., 2008a).

Whereas the fact that the SoA emerges from the interactive combination and integration of internal and external cues is corroborated by several behavioral studies (Moore & Fletcher, 2012), the sources of neural signals which permit external agency are yet to be determined. Brain regions involved in the motor system (the ventral premotor cortex, the supplementary motor area and the cerebellum) constitute a network for sensorimotor transformations and motor control, while heteromodal association cortices (the prefrontal, parietal and temporal cortex) are implicated in more conceptual cognitive processes (David, Newen, & Vogeley, 2008). Causal belief of agency changes the dynamics of sensorimotor networks, determining activity and connectivity modifications, with strengthened communication between the motor and temporal, parietal cortices and reduced participation of networks involved in predictive comparison process for mismatch detection (Buchholz, David, Sengelmann, & Engel, 2019).

Concurrently, abnormalities in sensory cortices are supposed to underlie the erroneous attribution (self‐triggered vs. externally triggered) of the action sensory feedback (Kikuchi et al., 2019; Martikainen, Kaneko, & Hari, 2005; Shergill et al., 2013). However, a basic sense of subjectivity is required for the high‐order conceptual attribution of the effect to the self (it is *I* who is causing it) (Barandiaran, Di Paolo, & Rohde, 2009; Salomon, 2017; Synofzik et al., 2008b; Tsakiris, Haggard, Franck, Mainy, & Sirigu, 2005). Indeed, disrupted SoA has been primarily ascribed to self‐related pathology with misidentification of the source of internally generated stimuli, that is, the agent (e.g., delusion of control in schizophrenia) (Frith, 2005; Frith et al., 2000; Keefe, Courtney, & McEvoy, 1997; Lindner, Thier, Kircher, Haarmeier, & Leube, 2005; Synofzik, Thier, Leube, Schlotterbeck, & Lindner, 2010).

Since the self is assumed to be purely based on internal processing (Boly et al., 2008; Gusnard, Akbudak, Shulman, & Raichle, 2001; Northoff, Qin, & Feinberg, 2011), it has been suggested that the brain's on‐going intrinsic activity (i.e., the resting‐state (rs) activity of the brain) may influence the implicit sensorimotor and high‐level conceptual processes leading to the causal self‐attribution of an effect (Robinson, Wagner, & Northoff, 2016). Crucially, the supposed correspondence between self‐referential and rs processes in certain regions of the brain, that is, the self‐rest overlap (Bai et al., 2016; Northoff, 2016a), is substantiated by recent fMRI (Davey, Pujol, & Harrison, 2016; Huang et al., 2015; Murray, Debbané, Fox, Bzdok, & Eickhoff, 2015) and neurophysiological studies (Wolff et al., 2019). Indeed, it has been suggested that the global spontaneous brain dynamics resulting from the temporally evolving patterns of interaction between different brain areas are directly related to the sense of self at a mental level. As the brain's rs activity can integrate neural activity over longer stretches of time, the integrative function of the self on the psychological level of SoA will be based on its temporal continuity. Such spatiotemporal continuity and integration would be manifest at both the neuronal and the mental level, thus providing the “common currency” of both brain and self (Northoff, Wainio‐Theberge, & Evers, 2019).

This paradigm shift moved research from the localization of functional areas relevant to self‐agency attribution to the investigation of large‐scale functional connectivity networks (Greicius, Krasnow, Reiss, & Menon, 2003) representing the neural architecture of the self (Gusnard et al., 2001). Thus, a new model of agency was proposed (Robinson et al., 2016) as to experimentally test the influence of ongoing rs cerebral activity in attribution JoA. It also allowed the scientific investigation of functional connectivity patterns‐unconfounded by processing of specific stimuli and cognitive demands‐relevant to self‐agency attribution. By defining the brain on the basis of its spontaneous temporo‐spatial dynamics rather than neurocognitive, this larger and more comprehensive framework enables the investigation of mental features (like the sense of self), which cannot be limited to the short and discrete moments in time and space as functions (Northoff et al., 2019).

Taking into consideration these premises, we aimed at exploring in healthy people, in the framework of network theory (Barabási, 2016), the relation between performance in an agency attribution task measuring self‐agency and brain rs‐functional connectivity. In particular, we aimed at unearthing the network of functionally related areas involved in information processing and integration sustaining self‐agency attribution.

It has been suggested that the positive sense of agency reflects the default state of brain networks associated to sensorimotor integration. Thus, we expected as a global property across subjects, centrality measures from network analysis to vary along with self‐agency experience within sensory and motor networks related to the online monitoring of sensorimotor signals (Cui et al., 2014).

## 2 MATERIALS AND METHODS

### 2.1 Subjects

Forty naïve healthy subjects (male *N* = 15) participated in this study. All subjects were carefully screened for a current or past diagnosis of any DSM‐5 Axis I or II disorder using the SCID‐5 Research Version edition (SCID‐5‐RV: First, Williams, Karg, & Spitzer, 2014) and the SCID‐5 Personality Disorders (SCID‐5‐PD: First, Williams, Benjamin, & Spitzer, 2016). Inclusion criteria were: (a) age between 18 and 65 years, (b) normal or corrected to normal vision (c) at least 8 years of education, (d) suitability for MRI scanning. Exclusion criteria included: (a) a history of psychoactive substance dependence or abuse during lifetime (b) a history of neurologic illness or traumatic brain injury with loss of consciousness, (c) major medical illnesses, that is, diabetes not stabilized, obstructive pulmonary disease or asthma, hematological/oncological disorders, B12 or folate deficiency as evidenced by blood concentrations below the lower normal limit, pernicious anemia, clinically significant and unstable active gastrointestinal, renal, hepatic, endocrine or cardiovascular system disease, newly treated hypothyroidism, (d) the presence of any brain abnormality and microvascular lesions apparent on conventional FLAIR‐scans. The presence, severity and location of vascular lesions was computed using the semi‐automated method recently published by our group (Iorio et al., 2013), (e) the presence of motion‐related MRI artifacts hindering data pre‐processing, (f) IQ below the normal range according to TIB (Test Intelligenza Breve, Italian analog of the National Adult Reading Test—NART–) (g) global cognitive deterioration according to a Mini‐Mental State Examination (MMSE) (Folstein, Folstein, & McHugh, 1975) score lower than 26, (h) major or mild neurocognitive diagnosis according to DSM‐5 criteria (American Psychiatric Association, 2013), (i) personality disorder, any present mental disorder and past major mental disorders (e.g., schizophrenia, bipolar disorder, major depressive disorder, etc.), (j) non‐Italian language native speaker, (k) color blindness as referred by the subject.

### 2.2 Neuropsychological and behavioral assessment

In order to assess the cognitive processes related to SoA (i.e., attention and executive functioning, [David et al., 2008]) and after having being screened for global cognitive impairment using the Mini‐Mental State Examination test (Folstein et al., 1975), all study subjects underwent a neuropsychological battery performed by a trained neuropsychologist. The Multiple Features Targets Cancelation test (Gainotti, 2001) evaluated selective attention abilities. The Trail Making Test parts A and B (TMT‐A and TMT‐B) (Reitan, 1992) were administered to appraise speed of information processing (TMT‐A) and set‐switching ability as a measure of cognitive flexibility and executive functioning (TMT‐B). The Stroop Word Color test (Stroop, 1935) was used to evaluate attention (word reading and color naming time) and cognitive flexibility (reading time for color words printed in incongruent ink). The Wisconsin Card Sorting test short‐version (Greve, 2001) evaluated executive processes. As to explore the potential relationship between SoA and psychological/evaluative aspects of the self (Gillihan & Farah, 2005), the Temperament and Character Inventory‐revised (TCI‐r) Italian‐version (Fossati et al., 2007) was administered. This self‐report questionnaire measures four temperament traits (harm avoidance, reward dependence, novelty seeking and persistence) and three character dimensions (self‐directedness, cooperativeness and self‐transcendence). Last, since it has been hypothesized that the SoA depends on a time window within which the signals related to the action and to the effect have to be integrated (Kawabe, Roseboom, & Nishida, 2013), subjects underwent a time‐color discrimination task (modified from Coull et al., 2011) in order to control for time perception and working memory abilities.

After applying exclusion criteria, two male subjects were excluded due to MRI artifacts and the total sample reduced to 38 subjects. All participants were right‐handed, with normal or corrected‐to‐normal vision. They gave written informed consent to participate after the procedures had been fully explained. The study was approved and carried out in accordance with the guidelines of the IRCCS Santa Lucia Foundation Ethics Committee. Data supporting the findings of this study are available on request from the corresponding author and not publicly available due to privacy or ethical restrictions. The study was founded by Italian Ministry of Health RC17, RC18, RC19 and 5Xmille 2018–2019 “Multidimensional study of timing abilities and sense of agency in schizophrenia and bipolar patients” and by the National Research Council (CNR) “A multifactorial intervention for successful aging” CUP J84I20000250005.

### 2.3 Experimental tasks

**FIGURE 1:**
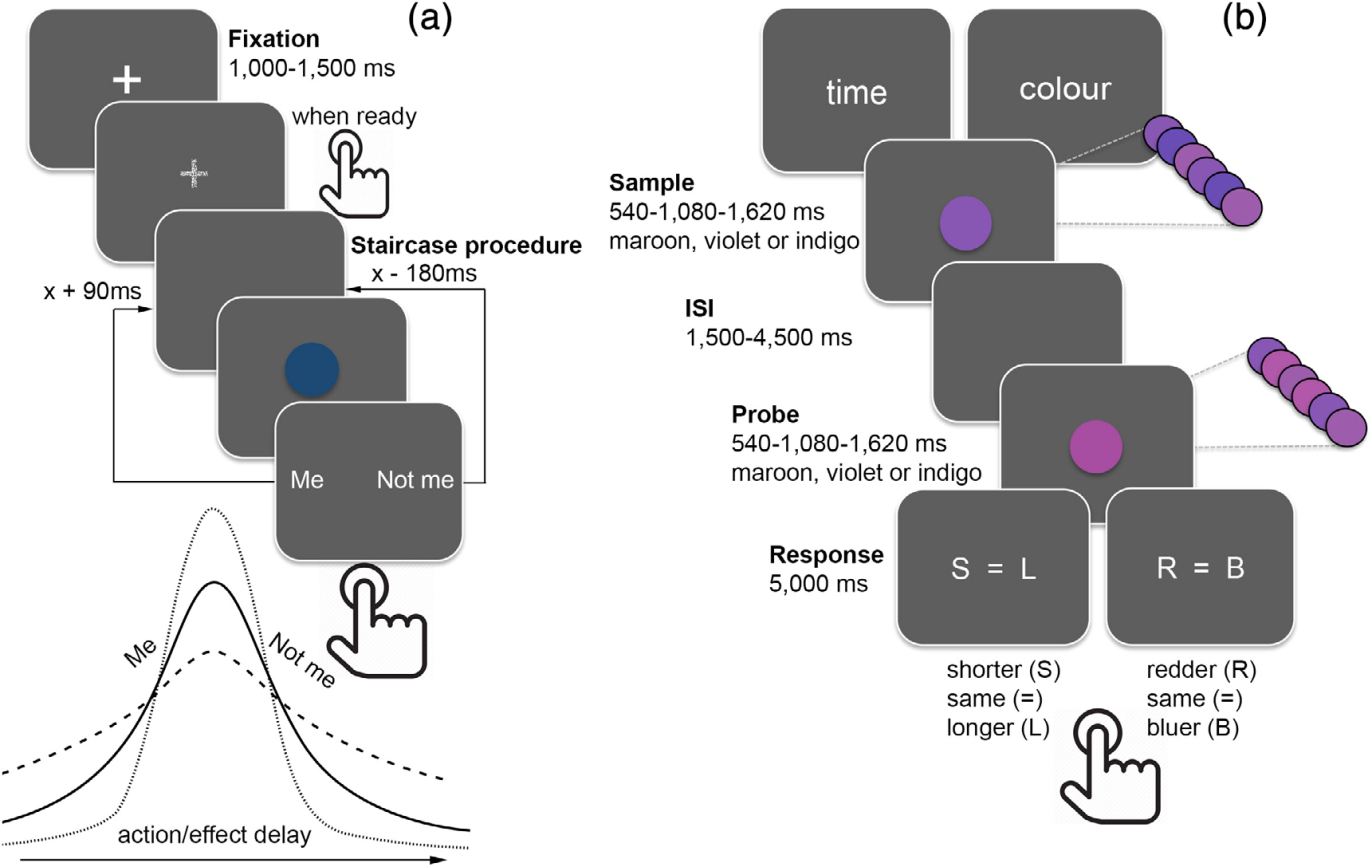
Experimental tasks. (a) Explicit Judgment of agency task. A key press (action) triggered the appearance of a blue ball (effect) after a delay (90–1,620 ms) varied according to a weighted up‐down psychophysical procedure where the delay was increased by 90 ms when subjects reported they caused the effect, and decreased by 180 ms when the symbol appearance was attributed to the computer. (b) Time and color discrimination task. In the time condition, participants estimated whether the duration of the second (probe) stimulus was shorter (S), equal to (=), or longer (L) than the previous (sample) stimulus. In the color condition, participants estimated whether the probe was redder (R), equal to (=), or bluer (B) than the sample. Participants estimated the average shade of purple (maroon, violet or indigo) by amalgamating all shades presented during rapidly alternating stimuli presentations (90 ms)

The experiment took place in two separate sessions. The two behavioral tasks (Figure 1) were administered in the same session with order of tasks counterbalanced across subjects. MR acquisition always preceded tasks administration within a 24 hr interval.

The *agency attribution task* measures participants' control judgments over a subsequent consequence of an action by asking to make an explicit attribution (“self‐triggered” or “externally triggered”) of a visual event. The sense of self‐agency here measured was experimentally manipulated by introducing visuomotor temporal incongruity (see below), and personally graded delay conditions were provided as to derive the individual time window for the action‐effect integration. The task was generated and stimulus presentation controlled using E‐Prime 2.0 (Schneider, Eschman, & Zuccolotto, 2002) on a Toshiba Satellite Pro R850‐1C8 with a screen refresh rate of 65 Hz. Participants were seated about 70 cm in front of the screen and required to fixate a white cross (duration randomly varying between 1,000 ms and 1,500 ms) at the center of a black background and to press with their non‐dominant hand the space bar, whenever they felt ready, once the fixation cross had disappeared. Since we were interested in measuring participants' explicit control judgments over a delayed visual event, the dominant hand was used to give the response, and reaction times in the judgment condition collected. The key press action triggered the appearance of a blue ball (2.06° in visual angle, 500 ms duration) at the centre of the screen. Figure 1a depicts the task timeline.

Immediately prior to the experimental task, participants underwent a training session of 20 trials, where the ball appeared 100 ms after the key press. Such training condition was meant to establish a strong causal relation between the key press action and the ball appearance.

In the following experimental task subjects were told that the ball appearance would be either caused by their own action or controlled by the computer when in fact, the ball appearance was always triggered by the subjects' key press. As we aimed at establishing the time window of agency attenuation (as an index of self‐agency), the delay between the key press and the ball appearance was systematically varied using two different staircase procedures: a descending one (70 trails with a starting delay of 1,620 ms) and an ascending one (70 trails with a starting delay of 90 ms) in a total of 140 trials. The delay range was chosen on the basis of previous reports (Farrer, Valentin, & Hupé, 2013) demonstrating a strong perceived control when the action‐effect delay was between 0 and 335 ms, and a loss of sense of control over the effect for delays longer than 708 ms. In each session the two procedures were interleaved and randomized in order to avoid participants' habituation to the experimental delay manipulation. In both procedures the delay was increased by 90 ms when subjects reported they caused the effect, and decreased by 180 ms when the symbol appearance was attributed to the computer. Such weighted up‐down psychophysical procedure, where the step upward (delay increase after a correct response) is half the step downward (delay decrease after an incorrect response) targets the 80.3% level on the psychometric function (Kaernbach, 1991).

On each trial, participants were required to judge, according to a two‐alternative forced choice: (a) “my space bar press directly triggered the ball”; (b) “the computer triggered the ball”, who was the agent that caused the ball appearance. We defined the first response “full control” and the second “no‐control”. Participants laid two fingers of their right hand on two keys of the numeric keypad (Key 1 and Key 2, respectively) and they had to respond as rapidly as possible by pressing one of the response keys.

The *time and color discrimination task* was administered to measure individual perceptual sensitivity in temporal perception and working memory abilities directly implied in the self‐agency indexes here quantified. Within the two (time and color) conditions the trial structure and stimuli used were identical, with the only difference being whether participants were asked to make time or color judgments (Coull et al., 2011). In the time condition, participants estimated whether the duration of the probe stimulus was shorter (S), equal to (=), or longer (L) than the previous sample stimulus. In the color condition, participants estimated whether the probe was redder (R), equal to (=), or bluer (B) than the sample. The response screen presented three alphanumeric characters, indicating each of the three possible responses for each condition. Participants pressed one of the three corresponding response buttons (left, middle or right) to indicate their estimate of relative duration or color. The response screen appeared for 5 s, during which the subject gave their response, and any response slower than 5 s was not recorded. Inter‐trial intervals varied pseudo‐randomly from 1 to 2 s. Figure 1b depicts both the time and color discrimination task. Participants performed 18 trials per condition, comprising an equal number of trials in which the probe was shorter than/longer than/equal to the sample in the time condition, or bluer than/redder than/equal to the sample in the color task. The time and color conditions were counterbalanced across two blocks of 18 trials, with each block comprising 9 trials of either the time condition or the color condition. The sample and probe stimuli were presented consecutively, separated by a pseudo‐random inter‐stimulus interval of 1,500–4,500 ms. Each stimulus was presented for one of three durations (540, 1,080 or 1,620 ms), and had an overall percept of one of three shades of purple (maroon, violet or indigo) ranging from a reddish to a bluish hue. Duration and color stimulus attributes were counterbalanced such that any of the three stimulus durations was paired with any of the three colors. The stimuli to be estimated were not a uniform color for the entire duration of stimulus presentation. Rather, rapidly alternating presentations (90 ms) of five different shades of purple across the entire stimulus duration gave an overall percept of maroon, violet or indigo. During the color task, the participant estimated the average shade of purple by amalgamating all shades presented during the flickering percept. This rather unusual color task has been designed to ensure that subjects maintain attention, and continually update their color estimate throughout the entire stimulus presentation. Such manipulation is crucial for equating sustained attention and working memory demands across the time and color tasks since time perception necessitates attention being sustained for the entire duration of the stimulus (~1 s), whereas perception of a static color would occur within the first 100 ms or so. Likewise, time perception requires stimulus onset to be held in working memory and then continually updated as time elapses, whereas perception of a static color would require no such updating of working memory.

### 2.4 MRI

rs‐fMRI data were collected using gradient‐echo echo‐planar imaging at 3 T (Philips Achieva) with a (T2*)‐weighted imaging sequence sensitive to blood oxygen level‐dependent (BOLD) (TR = 3 s, TE = 30 ms, matrix = 80 × 80, FOV = 224 × 224, slice thickness = 3 mm, flip angle = 90°, 50 slices, 240 volumes). A thirty‐two channel receive‐only head coil was used. A high‐resolution T1‐weighted whole‐brain structural scan was also acquired (1 × 1 × 1 mm voxels). Subjects were instructed to lay in the scanner at rest with eyes open. For the purposes of accounting for physiological variance in the time‐series data, cardiac and respiratory cycles were recorded using the scanner's built‐in photoplethysmograph and a pneumatic chest belt, respectively.

Several sources of physiological variance were removed from each individual subject's time‐series rs‐fMRI data. For each subject, physiological noise correction consisted of removal of time‐locked cardiac and respiratory artifacts (two cardiac harmonics and two respiratory harmonics plus four interaction terms) using linear regression (Glover, Li, & Ress, 2000), and of low‐frequency respiratory and heart rate effects (Birn, Diamond, Smith, & Bandettini, 2006; Chang & Glover, 2009; Shmueli et al., 2007).

rs‐fMRI data were then preprocessed as follows: correction for head motion and slice‐timing and removal of non‐brain voxels (performed using FSL: FMRIB's Software Library, www.fmrib.ox.ac.uk/fsl). Head motion estimation parameters were used to derive the frame‐wise displacement (FD), which in turn was used, together with its derivative, to correct data by a regression process. Time series were then demeaned, detrended, despiked and band‐pass filtered in the frequency range 0.01–0.25 Hz, using custom software written in Matlab (The Math Works). For group analysis, a two‐step registration process was performed. rs‐fMRI data were transformed first from functional space to individual subjects' structural space using FLIRT (FMRIB's Linear Registration Tool) and then non‐linearly to a standard space (Montreal Neurological Institute MNI152 standard map) using Advanced Normalization Tools (ANTs; Penn Image Computing & Science Lab, http://www.picsl.upenn.edu/ANTS/). Finally data were spatially smoothed (5 × 5 × 5 mm full‐width half‐maximum Gaussian kernel).

### 2.5 Statistical analyses

For *Agency Data analysis*, the number of “full control” responses was calculated at each temporal delay and normalized to the total number of trials. Number of correct responses, false alarms and missing responses was calculated for the time condition in the temporal and color discrimination task.

Agency attribution data were modeled using Matlab (The MathWorks) and color‐duration discrimination data were analyzed using R. In the former, for each participant, the normalized (to the total number of trials, i.e., 140) number of full control responses across the sampled delays was fitted to a Gaussian function. One participant was excluded from further analyses due to bad fitting results (*R* ^2^ = .23). Therefore, 37 subjects (mean age = 31.1, *SD* = 13.5; mean educational attainment = 15.6, *SD* = 2.6; male *N* = 12) composed the final study sample.

The delay corresponding to the curve peak value was taken as the Point of Subjective Equality (*t*_PSE_), representing the delay time at which probabilities for “full control” and “no‐control” responses are equal (50%). The curve peak value expressed how rapidly performance changed with changes in delay: the highest the value, the fastest subjects reached their PSE, being very consistent in their agency attributions. Indeed, the adopted psychophysical procedure increased the delay between the key press and the effect when the latter was attributed to the self. Therefore, small delays at which participants tended to have a full‐fledged sense of control over the ball were not shown again, while delays at which participants tended to experience a partial sense of control were repeatedly sampled. The curve SD expressed the range of delays at which uncertainty was high: the wider the curve, the strongest the temporal grouping between the action and its effect, as subjects tended to have a sense of control over the ball at both small and longer delays.

Crude correlations between the peak value of individual curves from the attribution agency data and accuracy from the time and color discrimination task, and between the peak value of individual curves, neuropsychological testing raw scores indexing attentional and executive abilities, and subscales scores from the TCI‐r were computed using the Fisher r‐to‐z transformation implemented in Statview. The same analyses explored the correlation between the SD of individual curves and time/color discrimination accuracy, neuropsychological testing raw scores and subscale scores from the TCI‐r. In order to control the expected proportion of incorrect rejections (Type I errors), significance was set at a False Discovery Rate (FDR) (Benjamini & Hochberg, 1995) = 0.05. Table 1 summarizes the derived behavioural indices.

**TABLE 1:** Agency attribution task: Investigated process and behaviorally derived indices

*Studied phenomenon*	
Sense of agency (SoA): The feeling that we are intentionally making things happen by our own actions	
Body agency	*I*'*m the cause or author of the movement* (e.g., The visual and proprioceptive experience that I'm in control of my action)
External agency (subtends body agency)	*I*'*m controlling my own action and through it, external events* (e.g., The feeling that I'm causing something to move) Self‐agency: *I take myself (and not someone else) as the agent of the effect caused my action* (e.g., When my intention is followed by an effect that resembles this content)
*Behavioral measures*	
Attribution JoA	Here measured as participants' explicit control judgment over a delayed (range 90–1,620 ms) visual effect (the appearance of a blue ball) Full control: *my space bar press directly triggered the ball appearance* No‐control: *the computer triggered the ball appearance*
Derived indices (agency attribution task)	Individual fitting to a Gaussian distribution of normalized (out of 140 trials) full control responses across sampled delays (based on a staircase procedure increasing the action‐effect delay when the latter was attributed to the self) *Mean curve peak delay* (action‐effect delay for 50% full control responses: Point of subjective equality, *t*_PSE_) *delay at which SS shifted from full control to no‐control responses* *Mean curve peak value* (proportion of full control responses at PSE) *consistency in attributing the effect to self at a certain delay* *Curve SD* (range of delays at which subjects tended to have a partial sense of control over the effect) *extent of the time window for self‐agency attribution*

In the *Network‐Agency analysis, rs‐*fMRI time series were averaged, for each participant, within 100 regions of interest (ROIs), combined in seven networks, and including cortical and sub‐cortical regions based on a rs‐and task‐based fMRI atlas characterized by functionally and connectionally homogeneous parcellations (Schaefer et al., 2018). A functional connection between two brain regions was assumed as an undirected and weighted graph link with the weight being the square of the correlation coefficient (Caldarelli, 2010). Here the squared correlation coefficients were considered as similarity indices (Goelman, Gordon, & Bonne, 2014), in order to account for the sign of correlations that result from neural‐mediated, temporally and spatially heterogeneous, hemodynamic mechanisms. The resulting matrix was thresholded by maintaining the graph fully connected, that is, implying that the number of graph components is equal to the graph size (de Pasquale, Della, Sprons, Romani, & Corbetta, 2016). *Degree centrality* (DC; Newman, 2010) and *clustering coefficient* (CC; (Watts & Strogatz, 1998) were used to characterize network topology in terms of connectedness and segregation at the local level. *Betweenness centrality* (BC; Newman, 2010) was assumed as a measure of the influence each region had over the flow of information within the network.

In order to find regions where the network topology measures were related to perceived control judgment across subjects a step‐wise regression approach was used. Network measures from the 100 brain regions as independent variables, and individual curve peak values as dependent measures were included in the analyses to investigate reciprocal dependencies that maximize the statistical explanation of perceived control over the effect. Network measures from the considered brain regions were also regressed on the SD of individual curves. So as to look at relationships without inflating the risk of a Type I error (Draper & Smith, 1998), and to increase the number of descriptors in the regression equation as to improve predictors' fit, a forward stepwise multiple regression model (*F* > 4 to enter) was chosen. Indeed, the forward stepwise procedure starts with no variables in the model and it tries out the variables one by one, including them if they are statistically significant, thus identifying the best set of predictors that gives the biggest improvement to the model. Simple linear regressions were preliminarily performed to include, in subsequent multiple regressions analyses, only variables significantly (*p* < .05) related to the behavioral measures considered. Multicollinearity between variables was tested by calculating the tolerance value of each viable predictor, that is, the proportion of variation in each predictor independent from the correlation between regressors (Berk, 1977). The cutoff value for including variables in multiple regression analyses was set such that the variability in a predictor not related to other variables in the model was at least larger than 30%. All statistics were performed on StatView statistical software.

## 3 RESULTS

### 3.1 Agency attribution task

At the group level, the mean curve peak delay (*t*_PSE_) was 625 ms (SD ± 288.52), while the mean curve peak value, was 0.21 (SD ± 0.06). Across subjects, variability within delays determining self‐agency attenuation was 16 ms. A significant negative correlation across subjects was found between the curve peak and the t_PSE_, (*r* = −.61; *z* = −4.97; *p* < .0001) as well as between the curve peak value and the curve SD (*r* = −.68; *z* = −4.97; *p* < .0001). No significant (using an FDR = 0.05) correlation was observed between individual curve peak values, SD of individual curves and neuropsychological testing raw scores indexing attentional and executive abilities. A significant negative correlation was observed between TCI‐r derived measures of the harm avoidance temperament dimension and the peak value of individual curves (*r* = −.393; *z* = −2.23; *p* = .025, FDR adjusted *p* = .041). A significant positive correlation was also observed between the peak value of individual curves and the personality trait of cooperativeness (*r* = .352; *z* = 1.97; *p* = .048, FDR adjusted *p* = .048). A significant negative correlation was additionally found between the SD of individual curves and the personality trait of self‐directedness (*r* = −.35; *z* = −1.96; *p* = .046, FDR adjusted *p* = .046).

### 3.2 Time and color discrimination task

Within an identical setup, participants were asked to make time or color judgments (Coull et al., 2011) by estimating whether the duration of a probe stimulus was shorter, equal to, or longer than a sample (temporal condition) or whether the probe was redder, equal to, or bluer than the sample (color condition). Data were available for a total of 34 studied subjects.

As for the temporal condition, mean accuracy in the study group was 84% (±0.1 SD) implying a very high sensitivity in discriminating the sampled temporal intervals. Mean accuracy in the color condition was 74% (±0.15 SD). No significant (using an FDR = 0.05) correlations were observed between time and color discrimination accuracy and values expressing how rapidly performance changed with changes in delay or variability within delays at which uncertainty was high. Table 2 reports demographic characteristics and behavioral results in the studied sample.

**TABLE 2:** Demographics and descriptive statistics for neuropsychological and behavioral tests

Demographics			
	*N* (mean)	*SD*	*Range (min–max)*
Gender. Male (%)	12 (32.4)	—	—
Age. Years	31.1	13.5	21–64
Education	15.6	2.6	8–21

*Neuropsychological and behavioral measures*	*Mean*	*SD*	*Range (min–max)*
TCI‐r novelty seeking	21	6.11	3–33
TCI‐r harm avoidance	14.16	7	1–31
TCI‐r reward dependence	14.34	4.6	1–23
TCI‐r persistence	4.57	1.55	1–8
TCI‐r self‐directedness	31.5	7.38	8–44
TCI‐r cooperativeness	32.5	7.27	8–40
TCI‐r self‐transcendence	12.69	6.74	1–28
MMSE	29.56	0.75	27–30
Stroop reading (s)	13.47	2.96	8–22
Stroop color (s)	17.07	2.91	12–23
Stroop interference (s)	25.15	5.68	17–38
Multiple features targets cancelation (s)	41.34	8.78	23–64
Multiple features targets cancelation recognitions (*n*)	12	1.67	7–13
TMT‐A (s)	39.62	13.5	2–70
TMT‐B (s)	72.03	28.61	35–175
TMT‐B‐A (s)	30.79	24.61	4–142
WCST category (*n*)	6	0	6–6
WCST perseverative errors (*n*)	0.06	0.35	0–2
WCST errors (*n*)	0.59	0.87	0–3
Discrimination task time accuracy (%)	0.84	0.1	0.61–1
Discrimination task color accuracy (%)	0.74	0.15	0.33–1

Abbreviations: MMSE, Mini‐Mental State Examination; TCI‐r, Temperament and Character Inventory‐revised; TMT, trial making test; WCST, Wisconsin Card Sorting Test.

### 3.3 Brain topological organization in self‐agency attribution

#### 3.3.1 Local connectedness, segregation and information flow related to perceived control over the effect

**FIGURE 2:**
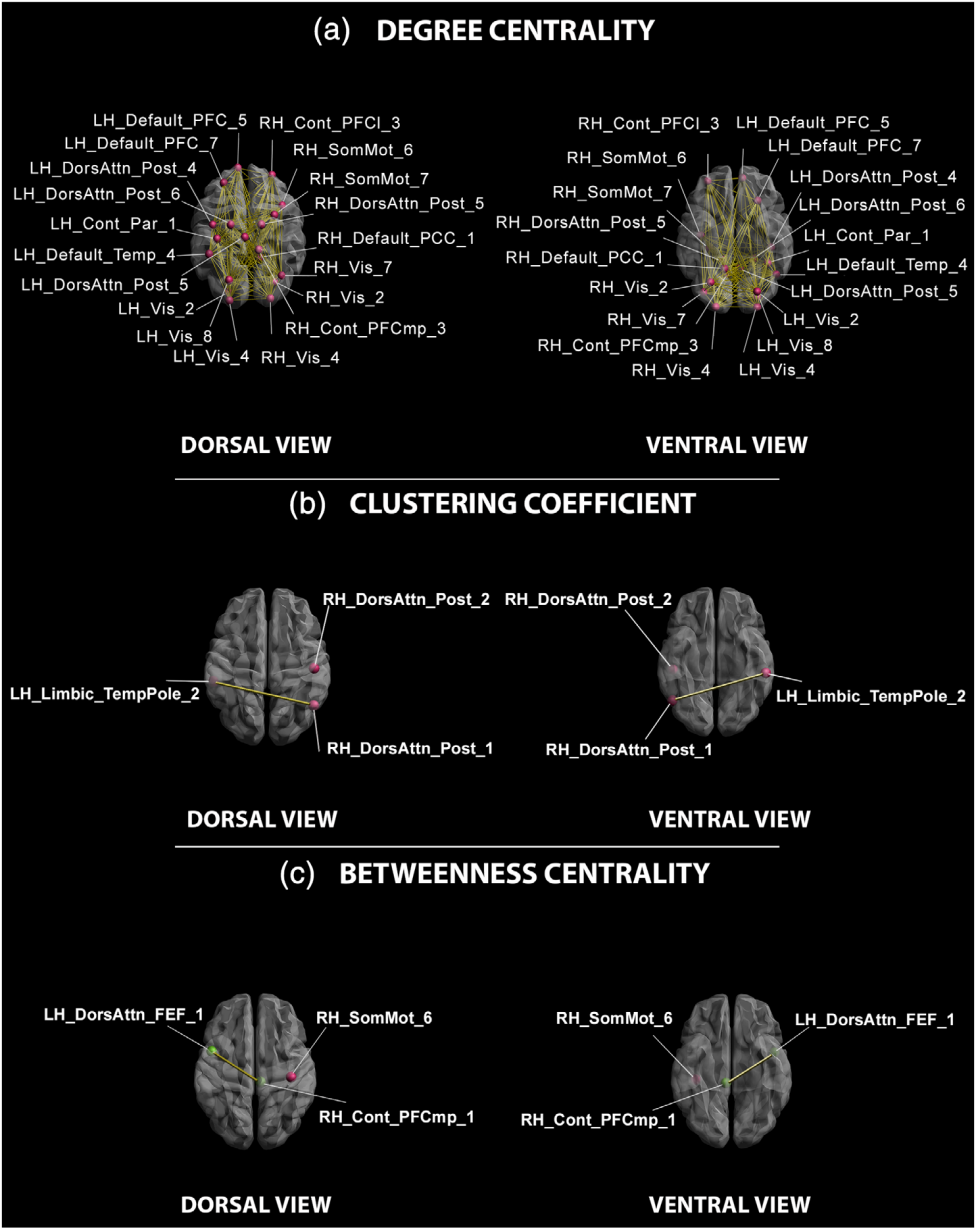
Nodes of cortical regions related to the curve pick values of subjects' performance in the Agency Attribution Task. 3D dorsal (left panel) and ventral (right panel) views of nodes resulted as significantly related to consistency in self‐agency attribution for: (a) Degree Centrality, (b) Clustering Coefficient, and (c) Betweenness Centrality indices. Red dots: nodes with positive relationships between centrality indices and behavioral measures; green dots: nodes with negative relationships between centrality indices and behavioral values; yellow lines: links between nodes significantly related to each other (*p* < .05). Labels from the Yeo and Schaefer Atlas, available from: https://github.com/ThomasYeoLab/CBIG/blob/master/stable_projects/brain_parcellation/Schaefer2018_LocalGlobal/Parcellations/MNI/Centroid_coordinates/Schaefer2018_100Parcels_7Networks_order_FSLMNI152_2mm.Centroid_RAS.csv. Cont_Par_1, First Parietal Control Network parcel; Cont_PFCl_3, third segment of the Lateral Prefrontal Cortex Default Network parcel; Cont_PFCmp_3, third segment of the Medial Prefrontal Cortex Default Network parcel; Default_PCC_1, Fifth segment of the Precuneal Default Network parcel; Default_PFC_5, Default_PFC_7, Fifth and seventh segment of the Prefrontal Cortex Default Network parcel; Default_Temp_4, fourth segment of the Temporal Default Network parcel; DorsAttn_Post_4, DorsAttn_Post_5, DorsAttn_Post_6, fourth, fifth and sixth segment of the Posterior Dorsal‐Attentional Network parcel; LH, Left Hemisphere; RH, Right Hemisphere; SomMot_6, SomMot_7, sixth and seventh segment of the Somatomotor Network parcel; Vis_2, Vis_4, Vis_7, Vis_8, second, fourth, seventh and eighth segment of the Visual Network parcel

The covariance between the explicit attribution judgment of self‐agency and the centrality of nodes within the whole functional connectivity network was investigated. Results from simple linear regressions showed significant positive correlations between curve peak values and the degree centrality in bilateral frontal, parietal and occipital regions, as well as between the curve peak values and the clustering coefficient in left temporal and right parietal and occipital cortices (see Table 3). Moreover, a significant covariance between the betweenness centrality of right cingulate and parietal, and left frontal cortical nodes and the curve peak value was found both as negative and positive correlations (see Table 3). Results from the subsequent stepwise regression showed that covariance between curve peak values and local connectedness (degree centrality) in the left occipital pole within the visual cortex explained 20% of observed variance. Further, the same analysis revealed that local segregation (clustering coefficient) within the right postcentral gyrus, part of the posterior portion of the dorsal attention network covaried with curve peak values, and such relationship explained 10% of the total variance. Last, the stepwise regression showed that covariance between curve peak values and the potential for control of information flow (betweenness centrality) in the left precentral gyrus, part of the dorsal attention network near the frontal eye field explained 26% of the total variance. Figure 2 depicts the cortical nodes related to the curve pick values of subjects' performance in the agency attribution task.

**TABLE 3:** Statistical results and MNI coordinates of nodes of cortical regions related to the curve pick values of subjects' performance in the agency attribution task

Cortical areas (Schaefer node label)	Beta	*R* ^2^	Adj *R* ^2^	*F*_(1;35)_	*p*	MNI centroids coordinates (Network; Schaefer et al., 2018)		
						*x*	*y*	*z*
*Degree centrality*								
Simple linear regressions								
L_Occipital fusiform gyrus (LH Vis 2)	.335	.126	.101	5.045	.031	−26	−76	−14
L_Occipital pole (LH Vis 4)	.469	.22	.197	9.85	.003	−26	−96	−4
L_Lateral occipital cortex (LH Vis 8)	.351	.123	.098	4.928	.033	−26	−88	20
L_Postcentral gyrus (LH DorsAttn_Post 4)	.332	.11	.085	4.345	.044	−42	−34	48
L_Precuneous cortex (LH_DorsAttn_Post 5)	.374	.14	.115	5.676	.023	−6	−60	56
L_Superior parietal lobule (LH_DorsAttn_Post 6)	.399	.159	.135	6.626	.014	−22	−50	66
L_Angular gyrus (LH_Cont_Par 1)	.328	.108	.082	4.225	.047	−38	−52	46
L_Lateral occipital cortex (LH_Default_Temp 4)	.34	.116	.09	4.571	.04	−48	−64	36
L_Frontal pole (LH_Default_PFC 5)	.364	.133	.108	5.362	.027	−8	48	42
L_ Superior frontal gyrus (LH_Default_PFC 7)	.389	.151	.127	6.227	.017	−26	20	52
R_Occipital fusiform gyrus (RH_Vis 2)	.401	.161	.137	6.692	.014	28	−66	−12
R_Occipital pole (RH_Vis 4)	.45	.203	.18	8.903	.005	22	−96	−4
R_Lateral occipital cortex (RH_Vis 7)	.426	.181	.158	7.751	.009	36	−82	16
R_Precentral gyrus (RH_SomMot 6)	.425	.181	.157	7.717	.009	40	−22	60
R_Postcentral gyrus (RH_SomMot 7)	.353	.125	.1	4.98	.032	30	−38	64
R_ Superior parietal lobule (RH_DorsAttn_Post 5)	.458	.21	.188	9.314	.004	14	−52	66
R_Frontal pole (RH_Cont_PFCl 3)	.333	.111	.086	4.372	.044	32	46	30
R_Precuneous cortex (RH_Cont_PFCmp 3)	.338	.114	.089	4.504	.041	10	−66	42
R_Precuneous cortex (RH_Default_PCC 1)	.326	.106	.081	4.164	.049	12	−54	14
Stepwise regression								
L_Occipital pole (LH_Vis 4)	.469	.22	.197	9.85	.003	−26	−96	−4
*Clustering coefficient*								
Simple linear regressions								
L_Inferior temporal gyrus (LH_Limbic_TempPole 2)	.343	.117	.092	4.658	.038	−58	−32	−22
R_Lateral occipital cortex (RH_DorsAttn_Post 1)	.358	.128	.103	5.154	.029	50	−62	16
R_Postcentral gyrus (RH_DorsAttn_Post 2)	.366	.134	.109	5.421	.026	50	−24	42
Stepwise regression								
R_Postcentral gyrus (RH_DorsAttn_Post 2)	.366	.134	.109	5.421	.026	50	−24	42
*Betweenness centrality*								
Simple linear regressions								
L_Precentral gyrus (LH_DorsAttn_FEF 1)	−.53	.28	.26	13.641	.001	−48	6	28
R_Precentral gyrus (RH_SomMot 6)	.337	.113	.088	4.471	.042	40	−22	60
R_Cingulate gyrus (RH_Cont_PFCmp 1)	−.37	.135	.111	5.482	.025	6	−28	34
Stepwise regression								
L_Precentral gyrus (LH_DorsAttn_FEF 1)	−.53	.28	.26	13.641	.001	−48	6	28

*Note:* Labels from the Yeo and Schaefer Atlas, available from: https://github.com/ThomasYeoLab/CBIG/blob/master/stable_projects/brain_parcellation/Schaefer2018_LocalGlobal/Parcellations/MNI/Centroid_coordinates/Schaefer2018_100Parcels_7Networks_order_FSLMNI152_2mm.Centroid_RAS.csv.

Abbreviations: Cont_Par_1, First Parietal Control Network parcel; Cont_PFCl_3, third segment of the Lateral Prefrontal Cortex Default Network parcel; Cont_PFCmp 1, Cont_PFCmp_3, first and third segment of the Medial Prefrontal Cortex Default Network parcel; Default_PCC_1, fifth segment of the Precuneal Default Network parcel; Default_PFC_5, Default_PFC_7, fifth and seventh segment of the Prefrontal Cortex Default Network parcel; Default_Temp_4, fourth segment of the Temporal Default Network parcel; DorsAttn_FEF_1, fifth segment of the Frontal Eye Field Dorsal‐Attentional Network parcel; DorsAttn_Post_1, DorsAttn_Post_2, DorsAttn_Post_4, DorsAttn_Post_5, DorsAttn_Post_6, first, second, fourth, fifth and sixth segment of the Posterior Dorsal‐Attentional Network parcel; L/LH, left hemisphere; Limbic_TempPole_2, second segment of the Temporal part of the Limbic Network parcel; R/RH, right hemisphere; SomMot_6, SomMot_7, sixth and seventh segment of the Somatomotor Network parcel; Vis_2, Vis_4, Vis_7, Vis_8, second, fourth, seventh and eighth segment of the Visual Network parcel.

As for the curve SD, significant covariance between the considered measure and degree centrality in left and right frontal, parietal and occipital regions was found as negative correlations (see Table 4). Simple regression analyses demonstrated a single significant positive correlation between the SD of individual curves and local segregation (clustering coefficient) in the right superior parietal lobule, part of the posterior portion of the dorsal attention network, and such relationship explained 13% of total variance. Significant covariance was also found in simple regressions between individual curves SD and betweenness centrality measures both as negative and positive correlations (see Table 4) in left frontal and occipital regions, and in a right parietal node.

**TABLE 4:** Statistical results and MNI coordinates of nodes of cortical regions related to the *SD* of subjects' performance in the agency attribution task

Cortical areas (Schaefer node label)	Beta	*R* ^2^	Adj *R* ^2^	*F*_(1;35)_	*p*	MNI centroids coordinates (Network; Schaefer et al., 2018)		
						*x*	*y*	*z*
*Degree centrality*								
Simple linear regressions								
L_Occipital pole (LH_Vis 4)	−.373	.139	.115	5.672	.023	−26	−96	−4
L_Frontal pole (LH_Default_PFC 5)	−.372	.138	.114	5.624	.023	−8	48	42
L_Precuneous cortex (LH_Default_PCC 1)	−.346	.12	.095	4.774	.036	−12	−56	12
R_Precentral gyrus (RH_SomMot 6)	−.345	.119	.094	4.721	.037	40	−22	60
R_Superior parietal lobule (RH_DorsAttn_Post 5)	−.334	.111	.086	4.392	.043	14	−52	66
R_Precuneous cortex (RH_Default_PCC 1)	−.33	.109	.083	4.277	.046	12	−54	14
Stepwise regression								
L_Occipital pole (LH_Vis 4)	−.373	.139	.115	5.672	.023	−26	−96	−4
*Clustering coefficient*								
Simple linear regressions								
R_Superior parietal lobule (RH_DorsAttn_Post 5)	.394	.156	.131	6.447	.016	14	−52	66
*Betweenness centrality*								
Simple linear regressions								
L_Occipital pole (LH_Vis 5)	.463	.215	.192	9.576	.004	−6	−92	−2
L_Precentral gyrus (LH_DorsAttn_FEF 1)	.536	.287	.267	14.096	.001	−48	6	28
R_Superior parietal lobule (RH_DorsAttn_Post 5)	−.382	.146	.122	5.981	.02	14	−52	66
Stepwise regression								
L_Occipital pole (LH_Vis 5)	.673	.453	.421	*F*_(2;34)_ = 14.09	<.001	−6	−92	−2
L_Precentral gyrus (LH_DorsAttn_FEF 1)						−48	6	28

*Note:* Labels from the Yeo and Schaefer Atlas, available from: https://github.com/ThomasYeoLab/CBIG/blob/master/stable_projects/brain_parcellation/Schaefer2018_LocalGlobal/Parcellations/MNI/Centroid_coordinates/Schaefer2018_100Parcels_7Networks_order_FSLMNI152_2mm.Centroid_RAS.csv.

Abbreviations: Default_PCC_1, fifth segment of the Precuneal Default Network parcel; Default_PFC_5, Default_PFC_7, fifth and seventh segment of the Prefrontal Cortex Default Network parcel; DorsAttn_FEF_1, fifth segment of the Frontal Eye Field Dorsal‐Attentional Network parcel; DorsAttn_Post_5, fifth segment of the Posterior Dorsal‐Attentional Network parcel; LH, left hemisphere; RH, right hemisphere; SomMot_6, sixth and seventh segment of the Somatomotor Network parcel; Vis_4, Vis_5, fourth and fifth segment of the Visual Network parcel.

**FIGURE 3:**
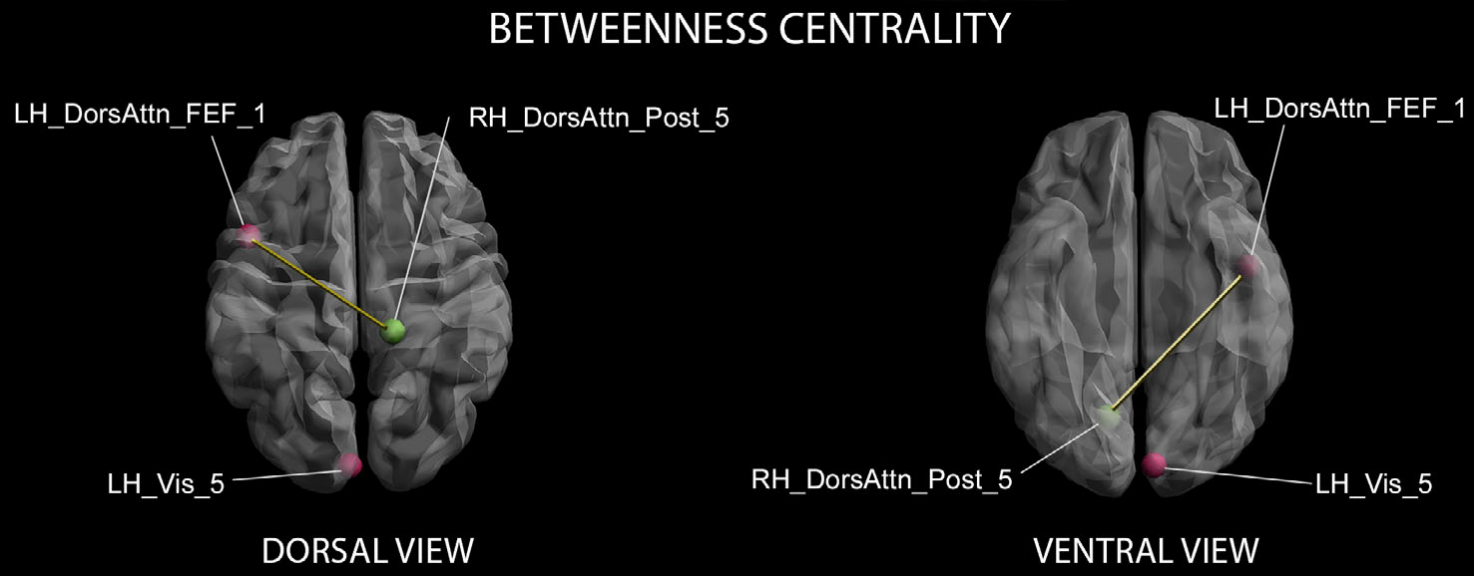
Nodes of cortical regions related to the SD of subjects' performance in the Agency Attribution Task. 3D dorsal (left panel) and ventral (right panel) views of nodes resulted as significantly related to the time window extent for self‐agency attribution considering Betweenness Centrality indices. Red dots: nodes with positive relationships between centrality indices and behavioral measures; green dots: nodes with negative relationships between centrality indices and behavioral values; yellow lines: links between nodes significantly related to each other (*p* < .05). Labels from the Yeo and Schaefer Atlas, available from: https://github.com/ThomasYeoLab/CBIG/blob/master/stable_projects/brain_parcellation/Schaefer2018_LocalGlobal/Parcellations/MNI/Centroid_coordinates/Schaefer2018_100Parcels_7Networks_order_FSLMNI152_2mm.Centroid_RAS.csv. DorsAttn_FEF_1, fifth segment of the Frontal Eye Field Dorsal‐Attentional Network parcel; DorsAttn_Post_5, fifth segment of the Posterior Dorsal‐Attentional Network parcel; LH, Left Hemisphere; RH, Right Hemisphere; Vis_5, fifth segment of the Visual Network parcel

Subsequent stepwise regressions showed a negative correlation between the curve SD and local connectedness in the left occipital pole within the visual cortex, and this relationship explained 11% of observed variance, while a complex of frontal and occipital nodes within the left dorsal attention and visual networks was the most influential in the information flow (betweenness centrality) related to dispersion of delays determining self‐agency attenuation, and such relationship explained more than 40% of observed variance. Figure 3 shows cortical nodes related to the SD of subjects' performance in the agency attribution task.

## 4 DISCUSSION

Here we map the brain topological organization related to information processing and signals integration essential for self‐agency attribution. From a behavioral point of view, we find that the time window for self‐agency attribution was 90–625 ms while feeling of control over the effect decreased for delays ranging from 658 to 1,600 ms. While no correlation was observed with measures of cognitive processes known to impact the SoA (David et al., 2008), personal beliefs about the self affected self‐agency, as subjects characterized by the tendency to be fearful, apprehensive and insecure have a broader action‐effect integration time‐window. At the neural level, we demonstrate that intrinsic brain processes linking somatosensory representations to incoming visual and proprioceptive information sustain individual consistency in attributing the effect to the self at a certain action‐effect delay. Conversely, extended self‐agency over long‐delayed effects is grounded on the intrinsic mode of brain function designed to organize information for visuomotor integration.

Indeed, connectedness at the local level in several cortical nodes within sensory (visual), somatomotor and association networks increases as consistency in the action‐effect integration at a definite delay increases. Specifically, the brain intrinsic functional organization sustaining consistency in the action‐effect integration at a certain delay is characterized by high connectiveness in the secondary visual cortex and by regional segregation in the primary somatosensory area. Motor and attentive control areas do not influence the information flow. Such functional network configuration, observable at rest, implies that a biological function, that is, the brain's on‐going intrinsic activity, is essential when action‐related perceptual and motor information have to be combined as to derive a feeling of control over external events.

This finding is highly informative for current models of SoA as it unearths the brain circuit organization underlying the conscious attribution of a delayed effect to one self's action. It also crucially demonstrates that self‐agency is grounded in the brain's intrinsic operations involved in the maintenance of information and representations central for interpreting and responding to the environment (Callard & Margulies, 2011; Raichle, 2015). It additionally substantiates the notion that the intrinsic mode of brain function is designed to organize information to register with changing regularities unfolding in the environment (Raichle, 2015). As a matter of fact, rs activity may correspond to continuous processing in the absence of stimuli or tasks, geared to conscious integration of information yielding adaptability, functionality and responsiveness to the challenges posed by the external reality (Mišić & Sporns, 2016).

Specifically, two sensory areas (i.e., the secondary visual and the primary somatosensory) are highly central and richly connected when consistency in the action‐effect integration at a certain delay is considered. Associative visual cortices have been previously implied in SoA, particularly in the self‐other distinction (Jeannerod, 2004) and related to intentional aspects of agency awareness. As the success of an action usually requires outcomes in the world, self‐agency greatly relies on the ability to visually monitor distal action effects. Moreover, the secondary visual cortex, parallelly processing motion, objects location and shape, is in the mainstream of the visual information flow related to visual‐motor integration. Indeed, the latter implies an intensive cross‐talk between cortical areas, since vision is used to plan movements in a feed‐forward fashion (Gallivan & Culham, 2015), whereas action‐specific plans influence visual feature processing (Gutteling, Park, Kenemans, & Neggers, 2013; Monaco, Chen, Menghi, & Douglas Crawford, 2018). The fact that this area showed the highest number of connections with other cortical nodes when measures of consistency in the action‐effect integration are considered, indicates that the state of preparedness and functional connectivity of this sensory region is crucial when competing internal and external cues have to be integrated as to derive a sense of control over external events. This observation fits well with the multifactorial account of SoA (Synofzik et al., 2008a; Vosgerau & Synofzik, 2012) assuming that all kinds of action‐related perceptual and motor information ‐and their congruence‐ are combined as to form an agency attribution. It also identifies the secondary visual area as the source of the external signals, which permit self‐agency attribution for delayed visual effects.

However, the observed clustered connectivity around the primary somatosensory area, which strengthened as the feeling of control over external events at a certain delay increased, suggests that a specialized processing allows the functional segregation necessary for integrating perceptual and cognitive states into explicit self‐agency attribution (Weiss, Tsakiris, Haggard, & Schütz‐Bosbach, 2014). Particularly, the intrinsic brain's operations sustaining perceived control over subsequent consequences seems to be mainly involved in the maintenance of somatosensory representations as to link them to incoming visual and proprioceptive information. Activity in somatosensory areas is necessary for the sensorimotor transformations and integration assumed by the comparator model (Blakemore, Frith, & Wolpert, 2001; Frith et al., 2000; Wolpert & Flanagan, 2001). However, the cue integration theory (Moore & Fletcher, 2012), posits that SoA comes about by many different agency cues, which are constantly weighted according to their reliability in a given situation. Evidence from perceptual research demonstrated that the brain often integrates its multisensory information in the same way, such that each cue is weighted according to its precision (van Dam, Parise, & Ernst, 2015). Therefore, the rich complex functional interactions (expressed by the high local clustering coefficient) here observed in the primary somatosensory area as a function of consistency in attributing the effect to the self, can be assumed as the neural basis of the cue integration mechanism for inferring self‐agency (Moore, Wegner, & Haggard, 2009). Actually, an area comprised within the dorsal attention network and involved in controlling visual attention activation as to re‐orient it in case of changes in visual stimuli, is the less influential over the flow of information when consistency in self‐agency is considered. This suggests that, when a delay in the visual effect is somehow expected, attention reorienting is unnecessary and areas devoted to it become less central in the information transmission.

Nevertheless, although we hypothesize that the time flexible self‐agency experience arises from the optimal multisensory integration occurring in a sensory area, this cue‐integrated SoA is probably not an instance of perception, but rather a composite of thought/judgment (Reddy, 2019).

Indeed, by systematically injecting delays between the action and its visual consequence, we find a broader sense of agency over the effect than previously reported (Farrer et al., 2013). Crucially to the present finding, external agency (Farrer & Frith, 2002) can be more flexible to temporal discrepancies than body agency. Other external cognitive processes such as the salience of the contingency, or personal beliefs about the nature of mechanisms mediating actions and effects, can dominate and overwrite the explicit judgment of control (Karsh, Eitam, Mark, & Higgins, 2016; Osumi et al., 2019; Wegner & Wheatley, 1999; Wen, 2019). Actually, we find that subjects characterized by the tendency to be fearful, apprehensive and insecure have a broader action‐effect integration time‐window. This indicates that previous beliefs about oneself impacted the explicit self‐agency attribution. Interestingly, in clinical populations characterized by an enhanced estimation of the self‐agency experience and inflated sense of responsibility (e.g., patients with obsessive compulsive disorder) (Gentsch, Schtz‐Bosbach, Endrass, & Kathmann, 2012; Rhéaume, Ladouceur, Freeston, & Letarte, 1995; Sookman, Pinard, & Beck, 2001) the aberrant sense of agency was related to the harm avoidance dimension. The exaggerated anticipation of negative consequences, and the dysfunctional belief that doing nothing to avoid them is equal to causing harm, may have led patients to think that outcomes were under their personal control (Belayachi & Van Der Linden, 2010; Salkovskis, 1996). Accordingly, in our subjects we find that the personality trait of self‐directedness is related to extended perceived control over the visual effect at increasing temporal visuomotor incongruence. Indeed, in individuals with high confidence in their own efficacy, and characterized by an internal locus of control (Rotter, 1966), uncertainty is restricted to a narrow range of delay. Once again, this observation confirms the hypothesis that previous beliefs of control and responsibility can influence different aspects of the self‐agency experience (Synofzik et al., 2008b), thus impacting explicit attribution JoA.

Intriguingly, the extended self‐agency experience is sustained by a specific brain intrinsic functional organization characterized by decreased connectiveness in the secondary visual area, as the time‐window for self‐agency attribution becomes broader, and by regional segregation in the superior parietal lobule. A network comprising the primary visual cortex and a region roughly centered on the frontal eye fields is the most influential over the information flow. Hence, an intensive cross‐talk between the primary visual area and regions within the dorsal attention network is needed for controlling, and possibly facilitating, the transmission of information relevant for sustaining the perceived control over delayed effects. Actually, the frontal eye fields are responsible for attentional orienting, visual awareness and enhanced perception (Moore, 2001; Vernet, Quentin, Chanes, Mitsumasu, & Valero‐Cabré, 2014). As they exert a top‐down modulation over ongoing visual processing (Bar et al., 2006; Silvanto, Lavie, & Walsh, 2006; Thompson, Biscoe, & Sato, 2005), the brain intrinsic activity within the network here observed is essential for sustaining detection and processing of long‐delayed visual effects. Moreover, extended self‐agency is segregated in a parietal region within the dorsal attention posterior network, an area known to contribute to explicit judgments about action consequences and involved in detecting deviations from expectancy (Kuhn, Brass, & Haggard, 2013). This implies that in subjects showing an extended temporal grouping between their action and the effect, less weight is attributed to internal signals. Therefore, cognitive inference of self‐agency is not based on the simple “readout” of low‐level indicators (Weiss et al., 2014), but rather on cognitive visuo‐attentional and visuomotor integration processes. Previous findings on the role played by multisensory integration mechanisms in body representation (Costantini et al., 2016) suggest that visuo‐proprioceptive integration is affected by the different temporal resolution of the proprioceptive system (Vroomen & Keetels, 2010). Moreover, the visual and sensorimotor systems have different sensitivity to asynchrony, as the partial match between intentions and proprioceptive feed‐back may overcome SoA, even though the visual feedback is delayed (Shimada, Qi, & Hiraki, 2010). It is therefore possible, that in order to extend the time‐window for self‐agency, the visuo‐attentional system prioritizes and facilitates the flow of visual information, as to increase the probability that it will be integrated in the explicit agency judgment. Multisensory integration processes for long‐delayed effects would then take place in the area closer to the sensory region receiving the visual delayed input (Shimada et al., 2010), that is, the parietal lobe, to which visual information is preferably transferred. However, inter‐individual differences in temporal sensitivity to multisensory integration (Costantini et al., 2016) may have determined the inter‐subject differences here observed in the action‐effect integration time‐window. Though no correlation was found between time discrimination accuracy and the extent of the time‐window for self‐agency attribution, the fact that temporal acuity is slightly different for filled (as in the temporal discrimination task) and empty (as in the agency attribution task) intervals cannot completely rule out this alternative hypothesis.

Before some concluding remarks, we have to acknowledge a few study limitations. First, the present investigation is limited to explicit aspects of the sense of agency, thus impeding any consideration on more implicit, unconscious factors (e.g., action selection, intentions, effort, etc.) influencing external agency. Nevertheless, the correlation here investigated between the brain topological organization at rest and the behavioral phenotype might have captured even low‐level processes involved in SoA. Indeed, the functional measure adopted is unconfounded by processing of specific stimuli and cognitive demands, as resting state networks are more “natural” and “core” with respect to the functional division derived from task‐evoked patterns of activity (Callard & Margulies, 2011). Second, the explored co‐variation between the brain organization at rest and explicit self‐agency attribution might be regarded as inherently exploratory. However, centrality measures provide connectivity maps within the entire gray matter, enabling investigation of highly distributed functions and emphasizing the collective organized operation of entire cognitive systems (Mišić & Sporns, 2016). Actually, network theory constitutes a useful framework in which to consider the brain in terms of its structure and function.

Additionally, the correlation between measures of resting state activity (when ‐by definition‐ no goal directed mental activity and minimal perceptive input are present) and behavioral performance is debated, and might be regarded as minimally informative respect to processes that are inherently task‐related. Nevertheless, the function here investigated presumes an inner‐oriented mental activity while the brain's intrinsic resting‐state activity has a preliminary influence to later processing of agency cues (Robinson et al., 2016). Since we intended to explore the relative contribution of internal and external mental contents on self‐agency attribution, including the brain resting state activity (Robinson et al., 2016), the adopted functional measure seemed the best methodological option. Moreover, the brain's spontaneous activity, and more specifically, its spatiotemporal dynamics, play a crucial role in yielding the respective mental content (Northoff et al., 2019), and exploring the rest‐stimulus interaction is crucial to the elucidation of the brain's contribution to self‐related processes (Huang et al., 2015; Northoff, Qin, & Nakao, 2010) including SoA. However, future studies comparing the brain's intrinsic activity to its extrinsic (i.e., task‐induced or stimulus‐induced) activity during agency attribution are warranted as to delineate the neural network contributing to the self‐agency experience in both states.

Finally, the studied sample was not numerous, and this constitutes an additional potential limitation of the present investigation. Nevertheless, the computed correlations between behavioral measures of perceived control over the effect and centrality indices always resulted in intermediate to large effect sizes (Cohen's *d* (Cohen, 1988) between 0.79 and 1.81; *η* ^2^ (Richardson, 2011) between 0.13 and 0.45), thus suggesting that the performed analysis was not underpowered.

To sum up, here we provide unique experimental evidence that perceived control over subsequent delayed effects is based on intrinsic brain processes that link somatosensory representations residing in the primary somatosensory area to incoming visual and proprioceptive information. Conversely, extended self‐agency over long‐delayed effects is grounded on the intrinsic mode of brain function designed to organize information for visuomotor integration. We also demonstrate that whereas the self‐agency experience arises from the optimal multisensory integration occurring in a sensory area, these internal non‐conceptual computations are available to other cognitive systems for further processing. Indeed, visuomotor integration processes and previous beliefs regarding the person impacted the explicit propositional representation of the self as an external agent.

These findings bear important clinical implications since activity attenuation in somatosensory cortices and aberrant resting‐state activity in self‐referential regions of the brain of individuals with schizophrenia have been shown to impact their attribution of agency (Robinson et al., 2016; Shergill, Samson, Bays, Frith, & Wolpert, 2005), being also related to current hallucinatory severity (Shergill et al., 2005). Additionally, the enhanced estimation of the self‐agency experience in patients with obsessive compulsive disorder was proven to be related to beliefs of control and responsibility (Belayachi & Van Der Linden, 2010; Salkovskis & McGuire, 2003). In this view, the observed positive association between resting state activity in somatosensory areas and explicit self‐agency attribution in healthy subjects indicates that self‐agency is sustained by a specific brain intrinsic functional organization. This paves the way for the clinical and experimental validation of this neuroscientific method in studying the phenomenology of agency and sense of self. The demonstration that psychological/evaluative aspects of the self influenced attribution JoAs in healthy subjects supports the hypothesis that external cognitive processes, such as personal beliefs, can dominate and overwrite the explicit judgment of control over external events. Such observation opens up the possibility for modulating the aberrant self‐agency experience in clinical populations by modifying dysfunctional beliefs or by altering the temporal structure of rs brain activity using neurophysiological techniques. This has crucial clinical relevance, especially in all the clinical conditions in which both rs activity and sense of agency are altered, including, but not limited to, schizophrenia. Indeed, recent theorizations already assumed that alterations in global brain's dynamics at rest underlie various psychopathological symptoms, thus suggesting that the brain's particular spatial and temporal structure at rest can be presumed as diagnostic or therapeutic markers in psychopathology (Northoff, 2016b, 2016c).

## CONFLICT OF INTEREST

The authors declare no potential conflict of interest.

## ETHICS STATEMENT

The study was approved and carried out in accordance with the guidelines of the IRCCS Santa Lucia Foundation Ethics Committee.

## PATIENT CONSENT STATEMENT

Subjects gave written informed consent to participate after the procedures had been fully explained.

## Data Availability

Data availability statement: data supporting the findings of this study are available on request from the corresponding author and not publicly available due to privacy or ethical restrictions.
